# Personal

**Published:** 1889-10

**Authors:** 


### Personal.

---

DR. JOHN B. ROBERTS, Professor of Anatomy and Surgery in the Philadelphia Polyclinic, has been chosen Professor of Surgery in the Woman's Medical College of Philadelphia, *vice* Dr. W. W. Keen appointed to Jefferson. Dr. Roberts is well known to the profession as one of the most accomplished of surgeons, as well as a most genial man, and we rejoice with his legion of friends at this appreciation of his skill as a surgeon and worth as a teacher.

---

SURGEON WILLIAM S. TREMAINE, U. S. A., until recently, on sick leave of absence in this city, was, under date of August 20, 1889, ordered, by direction of the Acting Secretary of War, to report in person to the Commanding General of the Department of the Missouri for assignment to temporary duty at the Post of Fort Leavenworth, Kansas.

Dr. H. Y. Grant has opened an office at No. 75 Niagara street, and will confine his attention to Ophthalmology and Otology. Dr. Grant is the son of Sir James Grant, of Ottawa, Ont., and we cordially welcome him to Buffalo.

Dr. Charles G. Stockton and Dr. Charles Cary, of this city, were elected members of the American Association of Physicians at its recent annual meeting in Washington, D. C.

Dr. Rollin L. Banta, of Buffalo, was elected Vice-President of the American Association of Obstetricians and Gynecologists for the ensuing year, at its recent annual meeting, held in Cincinnati, O.

Dr. Wm. C. Krauss, formerly of Attica, N. Y., and recently returned from an extended period of study in foreign universities, has opened an office at 176 Franklin street.

